# *dtool* and *dserver*: A flexible ecosystem for findable data

**DOI:** 10.1371/journal.pone.0306100

**Published:** 2024-06-25

**Authors:** Johannes L. Hörmann, Luis Yanes, Ashwin Vazhappilly, Antoine Sanner, Hannes Holey, Lars Pastewka, Matthew Hartley, Tjelvar S. G. Olsson

**Affiliations:** 1 Cluster of Excellence *liv*MatS, Freiburg Center for Interactive Materials and Bioinspired Technologies, University of Freiburg, Freiburg, Germany; 2 Department of Microsystems Engineering, University of Freiburg, Freiburg, Germany; 3 Earlham Institute, Norwich Research Park, Norwich, United Kingdom; 4 Institute for Building Materials, ETH Zürich, Zürich, Switzerland; 5 Institute for Applied Materials, Karlsruhe Institute of Technology, Karlsruhe, Germany; 6 MicroTribology Center μTC, Fraunhofer IWM, Freiburg, Germany; 7 European Molecular Biology Laboratory, European Bioinformatics Institute (EMBL-EBI), Hinxton, United Kingdom; 8 John Innes Centre, Norwich Research Park, Norwich, United Kingdom; Brigham Young University, UNITED STATES

## Abstract

Making data FAIR—**f**indable, **a**ccessible, **i**nteroperable, **r**eproducible—has become the recurring theme behind many research data management efforts. *dtool* is a lightweight data management tool that packages metadata with immutable data to promote **a**ccessibility, **i**nteroperability, and **r**eproducibility. Each dataset is self-contained and does not require metadata to be stored in a centralised system. This decentralised approach means that finding datasets can be difficult. *dtool’s* lookup server, short *dserver*, as defined by a REST API, makes *dtool* datasets **f**indable, hence rendering the *dtool* ecosystem fit for a FAIR data management world. Its simplicity, modularity, accessibility and standardisation via API distinguish *dtool* and *dserver* from other solutions and enable it to serve as a common denominator for cross-disciplinary research data management. The *dtool* ecosystem bridges the gap between standardisation-free data management by individuals and FAIR platform solutions with rigid metadata requirements.

## Introduction

The immense amount of data underlying today’s scientific research requires strict management principles. Over the last decade, the attributes **f**indability, **a**ccessibility, **i**nteroperability, and **r**eusability have become the key guiding principles of good data management [1]. Making data “FAIR” has since become the motivation behind countless data management efforts.

Often, the ultimate rationale for making data FAIR is making data AI-ready [2]. Yet, large parts of the scientific community do not benefit directly from AI-ready data. In these cases more direct motivators for FAIR data management are those that benefit the individual: increased visibility in the academic world, improved collaboration with both the future self and colleagues, and hence enhanced efficiency. Students and early-career researchers need general-purpose tools with low entry barriers that facilitate data documentation. Once data is documented, the transition to publishing data on large scale repository platforms becomes natural.

**Fig 1:**
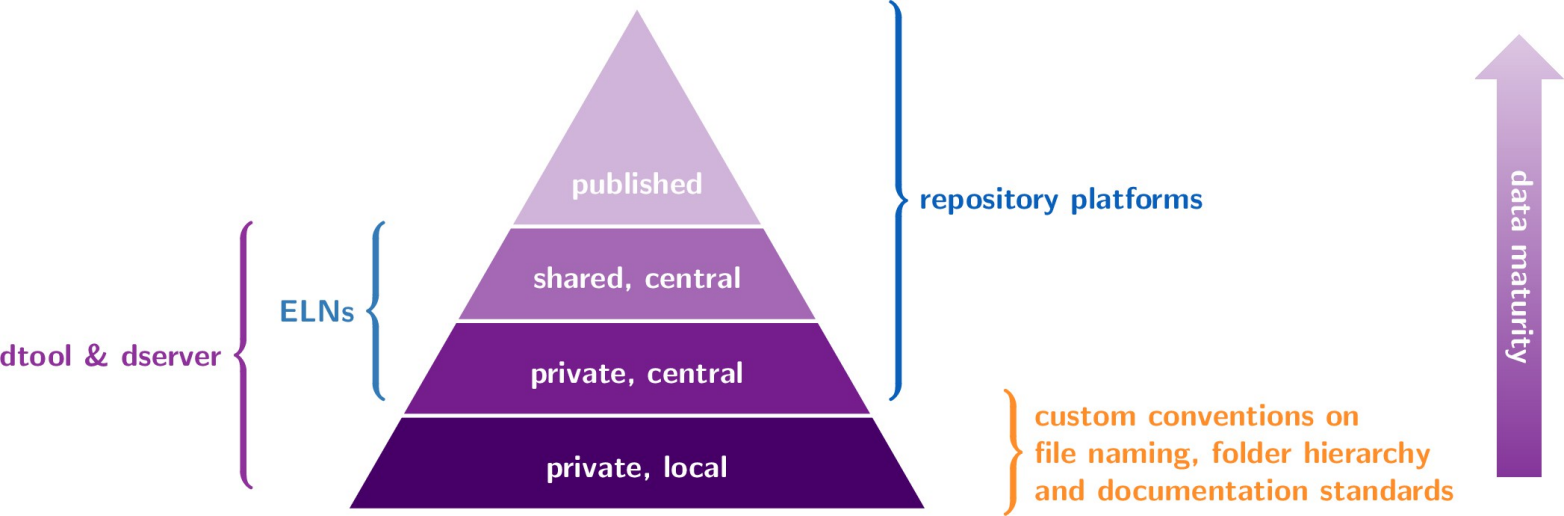
Data maturity pyramid. Scientific data evolves through several stages of maturity from initial collection towards publication. Different types of data management tools and platforms, indicated by curly braces, support the researcher along the stages of data maturity. Each of them operates on a different type of storage infrastructure, from hard drives in individual machines to object storage behind centralised public archives. *dtool* focuses on low data maturity where datasets are largely stored decentralised. *dserver* extends the scope of the ecosystem to an intermediate stage, where data is centrally archived and shared group-internally. ELN: Electronic Lab Notebook.

Lightweight data management with *dtool* as introduced by Olsson and Hartley [3] puts the focus on local data first and fills a gap (see Fig 1) between manual, naming-convention-based data management and complex discipline-specific, often cloud-based, integrated solutions such as centralised electronic lab notebook platforms (e.g. Chemotion [4]), repositories for computational data (e.g. NOMAD [5, 6]) or repositories for specialised metrology data (e.g. contact.engineering [7]). *dtool* exposes a Python API (application programming interface) and a command line tool to bundle data and metadata into a unified whole, referred to as a “dataset”. *dtool* also implements robust consistency checking to ensure integrity of datasets.

*dtool* is designed for operation in decentralised and highly distributed environments where needs differ substantially from group to group. This is a scenario where centralised solutions are difficult to implement and storage technologies change rapidly. By bundling data and descriptive metadata in datasets without imposing any discipline-specific constraints *a priori*, *dtool* encourages the use of machine-readable metadata formats for data documentation and allows templates adhering to specific schemas, but does not enforce either. The decision on the degree of compliance with FAIRness, in particular in terms of **i**nteroperability and **r**eusability, lies with each user and their choice of documentation standards. Hence, *dtool* and its datasets do not necessarily fulfil the FAIR principles in general, but they provide a didactic bridge, with a low barrier to entry, between completely standardisation-free data management and fully FAIR platform solutions with rigid metadata documentation.

The data and metadata within *dtool* datasets are made programmatically **a**ccessible via a command line interface (CLI) and a Python API. This programmatic access asks researchers to bring along a certain willingness for working on a terminal, but in turn empowers them to transition more easily from manual data management to semi- and fully-automated workflows. Illustrative examples can be found in machine learning research [8], solid mechanics [9–11], multiscale simulations [12, 13], and molecular dynamics simulations [14].

In summary, *dtool* improves accessibility, interoperability and reusability by packaging data and metadata as a unified whole and providing programmatic means with said data and metadata.

The *dtool* lookup server, or just *dserver*, described in this publication, makes *dtool* datasets **f**indable, hence rendering the *dtool* ecosystem fit for FAIR-completeness. It adds a centralised instance to the distributed data management ecosystem that makes dataset repositories searchable. Compared to *dtool* in isolation, *dserver* allows efficient querying of datasets indexed in a database via a standardised REST (representational state transfer) API to build suitable interfaces for both human interaction as well as machine interaction for workflow-integration. Their simplicity, modularity, accessibility and standardisation (via an API) distinguish *dtool* and *dserver* from other solutions and enable it to serve as a common denominator across interdisciplinary research data management (RDM). Abstracting away the storage layer allows quick and simple instantiation of searchable data repositories on the infrastructure at hand, be it a Windows share, an S3 object storage, or just a local file system. Brokers for other storage technologies are easily implemented and plugged into the ecosystem.

In this paper, we describe *dserver* and the decisions that lead to its design, and illustrate how it can benefit individual researchers and research groups. *dtool* and *dserver* are also contextualised by comparing it to other research data management solutions.

## Methodology

### *dtool* design

The core of good data management is data documentation [15]. Thus, bundling data and documentation into a unified whole lies at the core of decentralised data management ecosystems. *dtool* is no exception and the design decisions for the *dtool dataset* have been described in detail by Olsson and Hartley [3]. Importantly, the datasets hold administrative (such as username, date of creation, and file sizes) and descriptive (such as experimental conditions or simulation parameters) metadata in machine-processable plain text formats such as JSON (*JavaScript Object Notation*) [16] and YAML (formerly *Yet Another Markup Language*, now *YAML Ain’t Markup Language*) [17]. A dataset’s README.yml file contains descriptive metadata that should be formatted as machine-processable YAML. Plain text is not strictly forbidden, but the file extension strongly encourages the use of YAML. A dataset’s *manifest* holds structural metadata on all files contained within the dataset. S1 Fig illustrates an abstract *dtool* dataset together with examples on how to interact with it via the provided interfaces. Each dataset contains documentation on its own structure to make itself understandable even in the absence of any *dtool* software. In the following, we will refer to aforementioned descriptive and structural metadata as a dataset’s *readme* and a dataset’s *manifest* respectively.

*dtool* abstracts away the storage infrastructure layer. Its core is a set of atomic operations on datasets, like dataset creation or copying. *Storage brokers* are responsible for translating these atomic actions to actual operations on the underlying storage infrastructure of a specific *storage endpoint*. Within the *dtool* context, a *storage endpoint* is referred to as a *base uniform resource identifier (base URI)*. Examples of base URIs are,

- file:///path/to/repository,
- s3://some-bucket or
- smb://some-network-share.

The base URI consists of a *scheme* that determines the *storage broker*, in these examples file for the local storage, s3 for Amazon’s Simple Storage System API and smb for Microsoft Windows Server Message Block protocol, followed by a resource endpoint name like a server name or a location within the specific storage system.

Datasets are understandable in their raw representation on a specific storage system. Storage brokers are required to attach a simple textual description and a machine-readable structure documentation of the dataset representation specific to the storage infrastructure. For the example of hierarchical file systems, textual description and machine-readable structural documentation are found within the .dtool/README.txt and .dtool/structure.json files, respectively.

A dataset is globally identified by its universal unique identifier (UUID). Instances of the same dataset may exist at several base URIs. The consistency of a dataset across multiple instances is verifiable by hashes that are stored in the manifest and computed when a *prototype* dataset is made immutable by *freezing*. One instance of a dataset at a particular storage location is uniquely identified by its URI. This URI is composed of the base URI and a locally unique identifier, i.e. a local folder name file:///home/my-user/some-dataset or the UUID as a suffix s3://some-bucket/1a1f9fad-8589-413e-9602-5bbd66bfe675.

### *dserver* design

*dserver* ingests datasets at targeted base URIs and indexes them to make them searchable. What part of the dataset is made searchable in what way is adaptable without much effort. At its core, *dserver* allows free text search on administrative and descriptive metadata. Server-side plugins may extend ingestion and search mechanisms.

*dserver* needs an immutable core interface that lives on a slowly evolving technological layer. We choose a web server that serves HTTP/HTTPS requests. We draft a REST API adhering to the OpenAPI v3 specification [18] that defines *dserver* at its core irrespective of implementation details. Adhering to OpenAPI specification provides a way for automatically documenting and validating API requests both on the web interface and the internal interfaces across *dserver*. This prevents coding errors and provides stronger guarantees about how data is handled internally.

The architecture and design choices made during the creation of *dserver* were aimed at creating a system that is useful and sustainable. To make the system useful, it was designed to be easy to deploy and consume. To make the system sustainable, it was designed to be as simple as possible, yet flexible and extensible. Below are brief high-level descriptions of the key features of the system which are: a design resting on the three modular pillars of the core application, a search plugin and a metadata retrieval plugin; Python and Flask as the language and framework of choice; the delegation of authentication to third-party services; authorisation for searching and registering datasets on a per-base URI level; the dataset ingstion mechanism; free-text search on all available metadata; piecewise metadata retrieval.

#### Modular design

**Fig 2:**
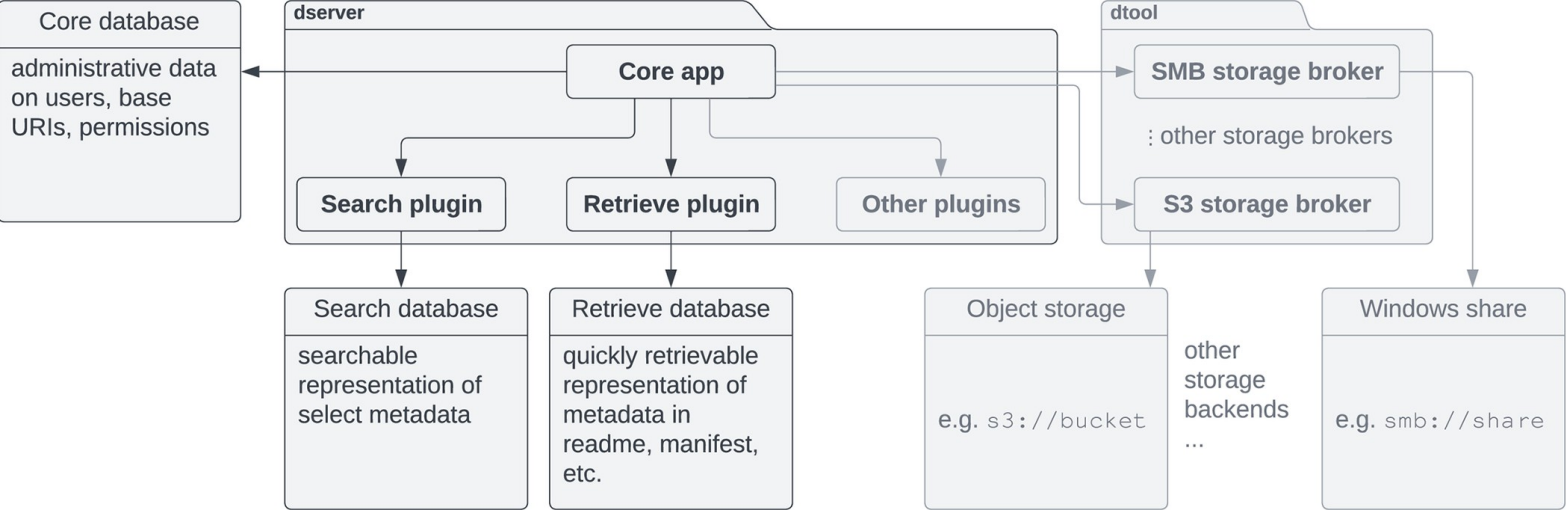
Generic components of a *dserver* instance embedded in a hypothesised environment. Arrows indicate dependencies and interaction. The *dserver* implementation is formally split into three components: a core app, a search plugin and a retrieve plugin. This makes *dserver* flexible in terms of database technologies. Each of the core components independently interacts with an underlying database deemed best suitable for its purpose. In a meaningful application case, *dserver* interacts with storage infrastructure holding datasets via *dtool* storage brokers to build its search index. Components of an exemplary environment providing S3 object storage and a Windows share are shown slightly greyed out.

We split our minimal *dserver* implementation into three components: the core application, the search plugin and the retrieve plugin. Conceptually, the core application exposes the consumable interface and manages privileges in a core database. The search plugin takes responsibility for building a searchable index of registered datasets. For this purpose, it may maintain its own database. The decision on which information is made searchable in what way lies with the specific search plugin implementation. The retrieve plugin takes responsibility for delivering metadata such as readme, manifest, or annotations for registered datasets efficiently on demand and may maintain its own database as well. Beyond these three core components, *dserver* supports arbitrary plugins that provide extended functionality, usually by introducing additional REST API routes. All interactions between *dserver‘s* components and *dtool* datasets happen via *dtool‘s* API and the specific storage brokers. This conceptual design illustrated in Fig 2 makes *dserver* agnostic in terms of the database technologies used. The plugin architecture alleviates the need to modify the core code when introducing new features and facilitates extending *dserver* for niche use cases with tailor-made plugins.

#### Language and framework

Python is a language with an extensive ecosystem of modules for data- and web-applications. This makes it easy to create a web API that can talk to a variety of databases. Furthermore, it is a popular language with many scientists. This means, an extension of the framework becomes possible even for scientists with only moderate software-development knowledge. For a lean implementation via Python’s web server gateway interface (WSGI) [19], we choose Flask [20] as the web application framework and flask-smorest [21] as the REST API framework with support for autogeneration of OpenAPI documentation. Authorisation is handled by flask-jwt-extended [22].

#### Authentication

Authentication is the process of verifying a user‘s identity. In *dserver*, authentication is implemented using JSON Web Tokens (JWT) [23]. The *dserver* implementation has the ability to create users and generate tokens for them. However, a feature of using JWT is that the token generation can be delegated to a third party, for example an academic institute’s central identity management system. *dserver* treats a user as authenticated as long as a valid JWT token is provided, even if the user has not yet been registered on the server instance. Such a user will, however, have no authorisation for any interaction with *dserver*.

#### Authorisation

The concept of authorisation is concerned with who can do what. The problem requires the management of relationships between entities, for example to answer questions like: “is user A allowed to search the metadata about the datasets stored in base URI X?”. Authorisation is built into *dserver* and works at the base URI level. Users can be granted permissions to search and/or register metadata about datasets in a base URI. The underlying technology for implementing authorisation is a relational database. The interface to the relational database is abstracted away using the *SQLAlchemy* object-relational mapper so any relational database supported by *SQLAlchemy* can be used [24].

#### Ingestion

*dserver* delegates ingestion to *registration clients* that interface with *dserver* either via its REST API, or, if integrated into the server application in form of a plugin, via its Python API. The extraction and provision of correct metadata lies within the responsibility of the *registration client*. *dserver* does not insist on having physical access to the actual dataset. This allows users with registration privileges to construct *dserver*‘s database in any fashion of their choice. Our *dserver* implementation comes with a simple server-integrated *registration client* that indexes all datasets on a specific *base URI*. For this, *dserver* of course needs to have direct accessibility to any ingested *base URI*.

#### Free-text searching

The ability to do free text searching on all the metadata of datasets within *dserver* is the key functionality of the system. There are many different database technologies that implement free text searching of documents. To allow the system to be flexible, and allow users to be able to employ their technology of preference, free-text searching is implemented using a plug-in architecture. Provided with this paper is an implementation created using *MongoDB* [25]. However, it would be trivial to create implementations based on different database technologies. Search results contain basic administrative metadata relating to the datasets identified, such as the UUID and URI.

#### Metadata retrieval

Metadata retrieval is separated from free-text searching to make sure that the bodies of responses from searches do not become too large. In other words searches only return responses with basic administrative metadata, not all the structural and descriptive metadata stored in dataset *manifest* and *readme* files respectively. The retrieval of *manifest* and *readme* metadata is also implemented using a plugin architecture. This allows the database technology for doing these actions to be different from that used for searching. However, the implementation provided with the paper also uses *MongoDB*. Metadata for a particular dataset are accessed using the dataset’s URI.

## Use-cases

### Data repository

The core use-case of a data management tool is to facilitate data storage. *dtool* and *dserver* are presently used for managing small to medium-scale (~ 100 TB, ~ 10,000 datasets) data repositories on S3 and SMB storage infrastructure in a variety of disciplines from plant science, bioinformatics, materials science and chemistry at the author’s institutions. Metadata is largely manually created by the user before freezing and uploading to the storage system. Large scale repositories have been migrated seamlessly between storage infrastructures (e.g. from SMB to S3) using *dtool*.

An emergent benefit of using *dtool* and *dserver* has been improved internal communication and sharing of data. UUIDs of datasets are easily communicated by email or on group chats. Datasets not shared in this manner are easily findable by other group members via the group-central *dserver*. In addition, embedding UUIDs in the comment sections of slide decks or manuscripts allows tracking data from the final products of scientific research, which are typically presentations or publications.

While *dtool* does not impose any metadata standards, some of us have formalised a metadata scheme for relationships between datasets. This gives a directed graph of datasets, that allows tracking the data processing pipeline from the raw data (e.g. a measurement or simulation input file) to various levels of derived data (e.g. interpretation of a spectroscopic experiment, simulation output, or a manuscript).

### Data transfer

Transferring large amounts of data between group members, institutions, or computer centres can be a challenge, and dedicated file-transfer solutions (e.g GridFTP [26]) have been created for various applications. *dtool* facilitates transfer in particular because it enables researchers to use modern web-based storage platforms such as Azure or S3 that have robust security mechanisms and are not hidden in private networks. For some of us, *dtool* has been instrumental in direct transfer of datasets from high-performance compute centres, collecting results of simulations run at various sites into a central location. On the S3 storage broker, *dtool* can also be used to share large datasets with outside collaborators by generating temporary URIs based on presigned-key mechanisms available in this system.

### Workflow automation

*dtool* datasets can be created programmatically through the Python API. This enables workflows that automatically generate *dtool* datasets and upload them to the storage system. This is particularly useful for complex simulations that need to be run for a variety of input parameters. These parameters are easily documented in the *readme* metadata and then become searchable within *dserver*. In combination with workflow management systems, this becomes a powerful ecosystem for automatically orchestrating simulations on high-performance computing systems. For example, we have used *dtool* and *dserver* in combination with the workflow system FireWorks [27] to orchestrate parametric runs of molecular dynamics simulations (see Ref. [14]). Analysis and postprocessing of these simulations then occurs through Python scripts that query simulation results for specific sets of parameters.

We are currently extending this system to decide which molecular simulation to run based on an active-learning scheme. *dserver* hosts the database that is used to train a nonparametric regression of a macroscopic constitutive law [12]. Retraining happens when a new dataset is uploaded to the storage system, and we decide the parameters of new simulation runs based on the estimated prediction error of the regression.

### Data management training

Discussions, and training material, around data management benefit from tools that can be used to illustrate its purpose and benefits. We have incorporated *dtool* and *dserver* into research data management workshops for junior researchers from an interdisciplinary spectrum of backgrounds. Trainings are based on the documentations for the *dtool* command line client [28], the *dtoolcore* Python API [29], and *dserver* [30]. Hands-on exercises illustrate a few core concepts of good research data management in a discipline-agnostic manner beyond conventions on file naming and folder hierarchy [31]. *dtool* and *dserver* force workshop participants to think about granularity and documentation when packaging data as datasets. It also introduces them to globally unique identifiers. When making their datasets findable through *dserver*, they come in touch with the benefits of machine-readable metadata above purely textual documentation.

## Discussion

Producing FAIR data is at the core of Open Science [32] and in the interest of research institutes, of science and society as a whole. For the scientific community, FAIR data means increased data quality, reproducibility, and verifiability. By extension this means enhanced efficiency, reduced redundancy, and reduced costs. For the individual, FAIR data should mean increased visibility in the academic world, improved collaboration with both the future self and colleagues.

Initially, however, improving data management standards is laborious and costly, and often these initial barriers hamper a change towards better practices. A system that supports data management should hence have a shallow learning curve and acknowledge the fact that FAIRness is a spectrum. Going FAIR is not a binary all-or-nothing choice and users should not be coerced dogmatically into going all the way at once.

*dtool* itself is installable by a single command and accessible to anyone with familiarity of Python and the command line. Installing and managing *dserver* requires a working knowledge of databases, specifically, in the case of the default setup, MongoDB and a relational database of choice. A working setup of *dserver* will also require access to a shared storage system for depositing datasets to be ingested. To extend *dserver* to handle alternative database technologies or add new functionality requires knowledge of Python programming.

*dtool* alone targets individual data management and encourages machine-readable data documentation. *dserver* extends the distributed *dtool* ecosystem by making the datasets in one or more base URIs searchable. The setup of a *dserver* can be done ad-hoc on storage infrastructure at hand. *dtool* datasets become findable at the desired level, whether group-wide, institutional, or global. Doing so moves the data a step closer towards true FAIRness.

The following overview embeds *dtool* and *dserver* within the current open-source landscape of research data management systems. The data taxonomy pyramid in Fig 1 correlates the quality (or *maturity*) of data with its storage location conceptually. It sorts data into four simple tiers [33]: data that is private and stored locally, data that is private but stored in some central location, shared data, and published data. Data starts privately at its creation. This data is often curated (and reduced in volume) while migrating to the shared and published tiers.

**Fig 3:**
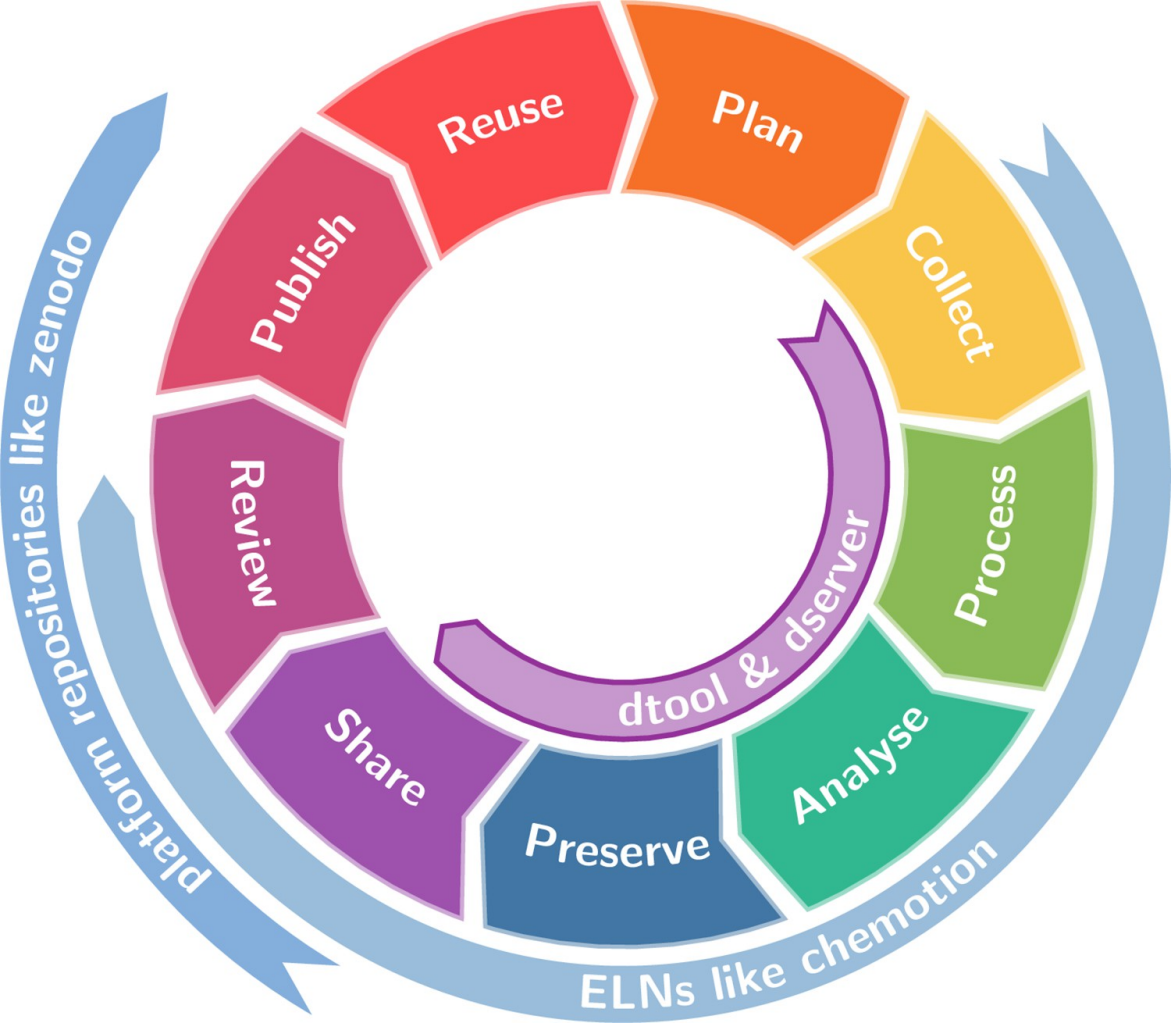
Data life cycle. Inspired by the ELIXIR Research Data Management Kit [34]. Scientific data evolves through subsequent stages, from planning and collecting to publication and reuse. Different types of data management tools and platforms, indicated by thin arrows, support the researcher along different segments of the data life cycle. *dtool* and *dserver* focus on the early stages from data collection to sharing, where data is handled by single individuals or within a research group.

The ubiquitous data life cycle in Fig 3 visualises the chronological evolution of data through subsequent phases in the course of an (academic) endeavour. The image of a cycle is not adequate in the sense that data does not just circulate repeatedly through a predefined flow, but evolves continuously. Nevertheless, the data life cycle is a widely accepted depiction of the chronological dimension in data evolution. We have extended the cycle by “Review” and “Publish” segments. In a generic scientific project kicked off with a planning phase, data is collected, processed, analysed, preserved, shared, reviewed, and published. Some of these steps may be skipped. If reused, the primary data enters another iteration of the cycle, leading to secondary data.

Specific data can be located at a unique point within the taxonomy pyramid in Fig 1 or within the life cycle in Fig 3, but no direct bijection between these two diagrams exists. While the life cycle captures the chronological evolution of data, the taxonomy pyramid captures their qualitative evolution. To understand how *dtool* and *dserver* fill a gap in the tool supply, it helps to identify which publicity tier or stage of life other research data management systems operate on.

General or discipline-specific platform solutions often come in pairs of a web-repository and an RDM framework. This is, for example, the case for general purpose repository zenodo.org [35] and its spin-off invenioRDM [36]. Clearly, such platforms target data at and beyond the transition from shared to published tier—or at a late stage in the data life cycle, as indicated by the annotation “repository platforms” within Figs 1 and 3. They are managed by independent organisations or on the institutional level.

Often discipline-specific electronic lab notebooks (ELNs) tackle a lower level in the taxonomy pyramid—or an earlier stage in the life span, as suggested with “ELN” annotations in Figs 1 and 3. An example are chemotion ELN and chemotion-repository.net [4]. Paired with a publicly accessible instance as in this case, they target data at and beyond the transition from private to shared data all the way to the published top. Ideally, raw data should enter ELNs at creation. Same as above, ELNs usually require central server instances, often managed on the department or institutional level, and hence cannot reach the local, truly private data at the bottom of Fig 1. Notably, recently developed PASTA-ELN [37] puts the focus on the lowest end of the data taxonomy pyramid in Fig 1 and may run locally, yet still requires a database instance.

Holistic approaches like openBIS [38] unite features of electronic lab notebooks and laboratory information management systems. They aim to capture the whole research data lifecycle within a single system. Consequently, this enforces modifications to existing processes and hence brings along the need for extensive staff training. Provision requires significant infrastructure and management resources, making sustainable instantiation and upkeep meaningful only on an institutional scope.

Contrary to above systems, *dtool* offers a distributed option for data management at the lowest level, where the responsibility for the format of data annotation rests with the user, as annotated within Figs 1 and 3. Together with *dserver*, the scope extends to the shared tier, making *dtool datasets* findable. This is possible on commodity storage infrastructure and with minimal administrative overhead, feasible even for groups with only a handful of members and a group-wide accessible network share. Yet, *dtool* and *dserver* span a scalable system that adheres to all applicable rules for workflow-readiness [39].

To understand the advantages and limits of *dtool* and *dserver*, we compare with other dataset-like digital objects. In an earlier publication [3], *dtool* was compared against the *BagIt* standard [40]. The major conceptual difference between *BagIt* and *dtool* is that *dtool* defines an abstract interface to data and metadata of a dataset in the form of an API, while *BagIt* is a specification for data and metadata storage on a classical file system. This difference applies in the comparison to other dataset-like standards as well. Other file system layout standards next to *BagIt* are *Moab* [41] and the *Oxford Common File Layout (OCFL)* [42], with the key goal of making a data repository rebuildable from the *OCFL* file system root without any further information. *BagIt*, *Moab* and *OCFL* reflect an evolution in the effort of making digital preservation sustainable at the low level of organising data and metadata on a classical file system. In *dtool*, this falls into the responsibility of the file system storage broker. The layout on a standard file system is described by Olsson and Hartley [3]. *BagIt*, *Moab* and *OCFL* were proposed in this chronological order by a community of institutional and preservation repositories with a focus on sustainable long-term archival of large volumes of data. The typical use case is under-the-hood in a platform solution. Here lies a fundamental difference to the origin of *dtool* and its datasets, which started with the idea of helping individuals with their distributed data management.

*DataLad* [43] is a tool motivated by the technical challenges of distributed data management. Built as a thin layer on top of git [44] and git-annex [45], it takes inspiration from established code management practices and puts a heavy focus on versioning. *dtool* and *DataLad* behave similarly in providing a command line interface (CLI) and a Python API at their core. *DataLad* covers a broader scope of features. Importantly, *DataLad* captures workflows and provenance next to data itself. Yet, to our knowledge no direct equivalent to *dserver* exists in the *DataLad* ecosystem. The DataLad-Registry project [46], however, comes close as it introduces benefits of centrality to the *DataLad* ecosystem and builds a catalogue of *DataLad* datasets publicly available on the web [47].

*RO-Crate* [48] addresses both the individual researcher as well as digital repository managers and infrastructure providers. Of all open-source RDM systems we are aware of, *RO-Crates* and the affiliated tool ecosystem are most suitable for direct comparison with *dtool* datasets and *dserver* in the sense that they comprise both a core specification for a dataset-like digital object that bundles data and metadata, the *RO-Crate* (Research Object Crate) [49], as well as libraries and tools for manipulating these objects. Arkisto [50] constitutes a repository platform built on top of *RO-Crates* and *OCFL*—in the purpose of providing a searchable collection of digital objects not unlike *dserver*. *dtool* datasets encourage YAML-formatted metadata, but essentially allow data documentation of any syntax, even plain text data. *RO-Crates* use JSON-LD and require adherence to the Schema.org dataset type [51] per specification. The more stringent documentation approach of *RO-Crates* certainly supports machine-readability better. JSON-LD [52] as a concrete Resource Description Framework (RDF) [53] syntax, for example, allows linking to external data and hence embedding metadata in ontologies, an important requirement for FAIRness. We argue that this apparent advantage of *RO-Crates* in terms of FAIRness and hence “machine-friendliness” comes at the cost of “human-friendliness”. *RO-Crates* fulfil their purpose as building blocks for data repositories well and could possibly become a standard format for FAIR digital objects [54]. They will, however, appeal less to the group leader who wants to improve their research data management without thoughts on semantic interoperability.

*OCFL*, *dtool*, *RO-Crates* and *DataLad* all bundle data and metadata but put their focus on different challenges of data management. The *OCFL* specifications [55] target completeness, parsability, versioning, robustness, and storage diversity on storage systems that present their data in a hierarchical manner. **Completeness** refers to datasets being a self-contained unit of metadata and content. A dataset needs to be understandable from their raw representation on the underlying storage infrastructure in the absence of any mediating software layer. **Parsability** refers to the ability of both humans and machines to understand the layout and inventory of a dataset. **Versioning** refers to the ability to incrementally change metadata or the content of a dataset and to track those changes. **Robustness** against errors, corruption and manipulation usually involves a mechanism for verifying the validity of an immutable dataset. **Storage diversity** refers to the ability to store datasets on a wide variety of storage technologies. The key features of *dtool* datasets overlap with *OCFL* in terms of completeness, parsability, and robustness. Versioning is deliberately excluded, but *freezing* a dataset, making it immutable, essentially makes versioning unnecessary. A new version in the *dtool* ecosystem would simply be a new dataset. Contrary to hierarchy specifications, *dtool* defines a dataset by API and hence carries the storage layer abstraction further, widening the scope of storage diversity. The foundation of *RO-Crates* lies in the principles of Linked Data [56] and hence they focus on parsability by standardising rich metadata and the semantics of data. Other aspects like completeness and robustness are delegated to the likes of BagIt or OCFL [55]. *DataLad* datasets put the focus on versioning—they are essentially git repositories. Table 1 compares how OCFL, *dtool*, *RO-Crate* and *DataLad* behave when regarded under the aspects of completeness, parsability, versioning, robustness, and storage diversity.

**Table 1:** Delineation of four distributed RDM concepts by their characteristics on the atomistic dataset level.

aspect	OCFL	dtool & dserver	RO-Crate	DataLad
**completeness**	Recommends self-containedness and self-description.	Administrative and structural metadata always bundled with content in the dataset. Documentation with descriptive metadata encouraged.	Recommends self-containedness and self-description.	Built on top of git and git-annex.
**parsability**	Both humans and machines can understand the layout and corresponding inventory regardless of software and infrastructure used.	Storage brokers create machine and human-readable structural descriptions of dataset representation. Structured descriptive metadata recommended, but not enforced.	JSON-LD and Schema.org Dataset mandated for metadata.	Delegates metadata handling to extension MetaLad, which automatically extracts metadata from a dataset and its contents by means of “extractors”.
**versioning**	Offers sophisticated versioning scheme	Data is immutable, metadata can be updated.	Offers sophisticated provenance tracking scheme.	Built on top of git and git-annex.
**robustness**	Checksums in inventory.	Checksums in manifest.	No built-in mechanism for data integrity verification.	Built on top of git and git-annex.
**storage diversity**	Designed for hierarchical storage systems.	Storage brokers allow the use of arbitrary storage infrastructure.	Assumes classical hierarchical file system.	Inherits limitations of git and git-annex. git-annex special remotes allow the use of arbitrary storage infrastructure.

Background coloring in red, yellow, or green indicates the degree to which the different concepts fulfill the aspects of completeness, parsability, versioning, robustness, and storage diversity. Note that these colors do not constitute a quality metric. The non-fulfillment of certain aspects often is a desired feature omission, such as the binary freezing mechanism for *dtool* datasets in place of incremental versioning.

## Summary & conclusions

*dtool* helps researchers to package data and metadata into a unified whole, the dataset. *dtool* datasets encourage systematic data documentation even for local distributed data, long before sharing or publication on a centralised repository. *dserver* makes *dtool* datasets searchable.

In comparison to other distributed data management ecosystems, *dtool* and *dserver* favour simplicity over features. They aim to make data management as painless as possible, whilst offering benefits of programmatic data access up front. Groups, labs, or institutes not yet provided with adequate RDM solutions through their organisations, disciplines, or communities, but in need for FAIRer data management solutions with little administrative overhead may benefit from the *dtool* ecosystem. Similarly, anyone overwhelmed by the endeavour for FAIR data and challenged by a rapidly changing infrastructure and services environment may find it a lightweight option for self-sufficient data documentation and archival. At the same time, human-accessible and programmatic interfaces allow seamless integration into any kind of workflow, from completely manual to fully automated examples.

*dtool* and *dserver* encourage the use of machine-readable metadata formats for data documentation and allow offering fillable templates adhering to specific schema, but do not enforce either. While the contents of a dataset are immutable, its metadata can be changed and therefore evolve with changes in requirements. Eventually, the decision on how far to go in terms of FAIRness lies with each user community and their choice of documentation standards. This distinguishes *dtool* and *dserver* from other data management ecosystems that either build conceptually around the strict adherence to standardized metadata schemas (e.g. *RO-Crate*) or exclude the handling of metadata from their core and delegate this task to extensions (e.g. *DataLad*).

To extend *dtool* and *dserver* to a fully FAIR ecosystem, one could develop a plugin for *dtool* that validates and enforces the use of metadata schemas. Similarly, one could create a plugin for *dserver* to validate that the metadata of ingested datasets adheres to specific schemas.

With *dserver*, a system administrator or data steward may create productive general purpose repositories on existing storage infrastructure, which can be a local file system, S3, Azure storage, or Windows share, with little configurational effort. Data management advocates in need of a complete RDM ecosystem for education purposes, i.e. for the duration of a workshop, find a quick and simple demonstrator solution in *dtool* and *dserver*.

As to be expected, just as with other RDM solutions the pay-off comes in the long run. The effort invested in deploying *dtool* and *dserver*, however, is scalable depending on the needs of the specific user group.

## Supporting information

S1 Fig *dtool* & *dserver* cheat sheet. Examples on how to inspect and, where applicable, manipulate metadata and data of a *dtool* dataset with the *dtool* command line interface (CLI), with the underlying *dtoolcore* Python API, and with the OpenAPI-compliant REST API of *dserver* for ingested datasets. These examples illustrate how *dtool* and *dserver* embed themselves well within the concept of workflow-ready software visualized in Fig 1 of Ref [39]. The right-hand side block shows the components of an abstract *dtool* dataset: descriptive metadata, administrative metadata, structural metadata, and the actual data. Descriptive metadata offers three levels of increasing complexity: tags, annotations (key-value pairs), and free-format YAML.
(TIF)
