# Supplementary figures and images for: dtool and dserver: A flexible ecosystem for findable data
Source: PLoS One. 2024 Jun 25;19(6):e0306100. doi: 10.1371/journal.pone.0306100 (PMC11198902; doi:10.1371/journal.pone.0306100)

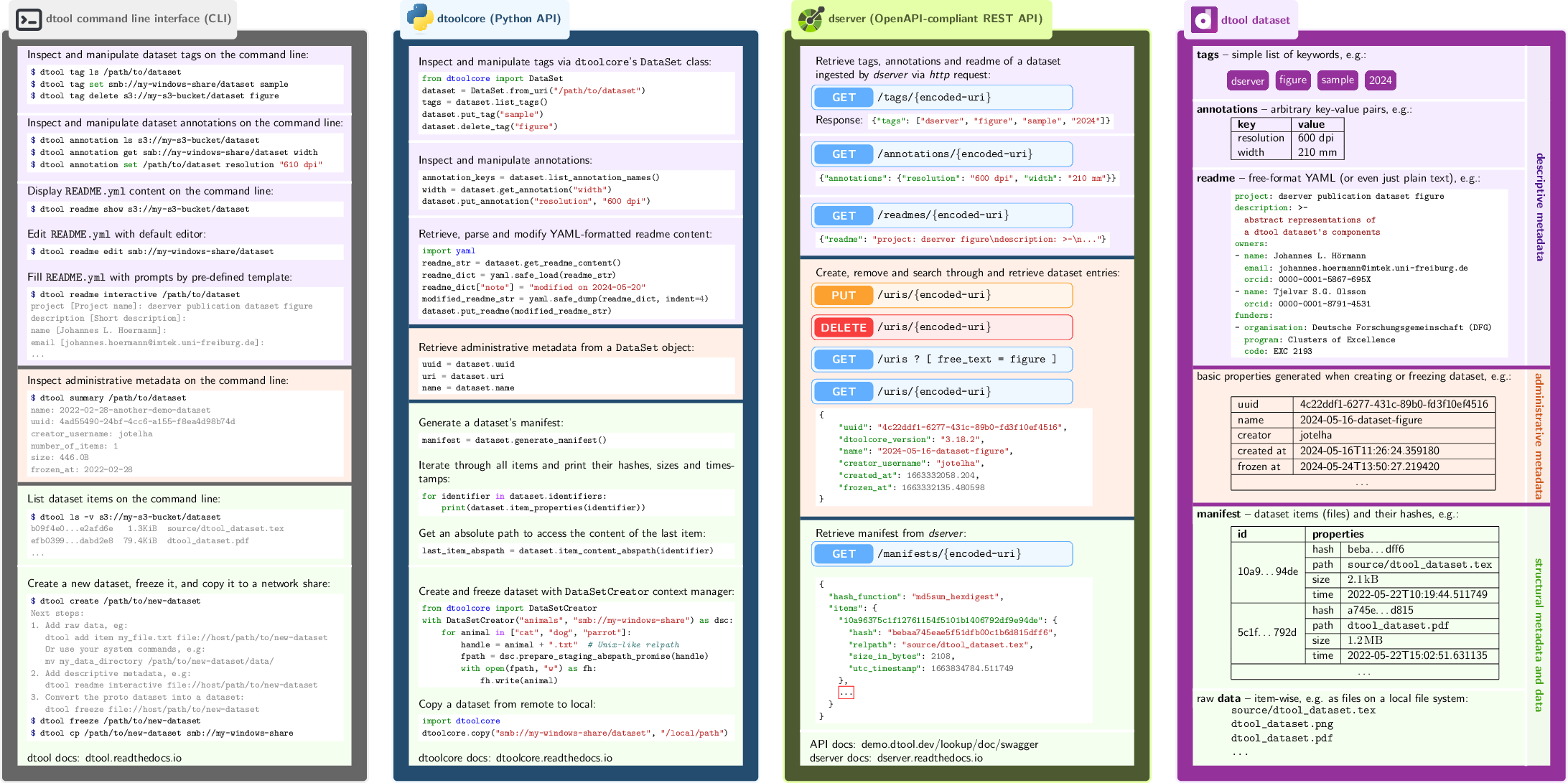

Supplement: S1 Fig — Examples on how to inspect and, where applicable, manipulate metadata and data of a dtool dataset with the dtool command line interface (CLI), with the underlying dtoolcore Python API, and with the OpenAPI-compliant REST API of dserver for ingested datasets. These examples illustrate how dtool and dserver embed themselves well within the concept of workflow-ready software visualized in Fig 1 of Ref [39]. The right-hand side block shows the components of an abstract dtool dataset: descriptive metadata, administrative metadata, structural metadata, and the actual data. Descriptive metadata offers three levels of increasing complexity: tags, annotations (key-value pairs), and free-format YAML. (TIF) [file pone.0306100.s001.tif]
